# Extraintestinal Invasive *Escherichia coli* Infections in the US

**DOI:** 10.1001/jamanetworkopen.2025.57201

**Published:** 2026-02-02

**Authors:** Heather N. Grome, Joshua M. Brandenburg, Alyssa G. Kent, Latasha Curtis, Ruben E. Raymond, Uzma Ansari, Amy S. Gargis, Susannah L. McKay, Erin Parker, Jennifer Driscoll, Karlie Hoetzer, Helen Johnston, Daniel R. McKenzie, Paulina A. Rebolledo, Emily Luckman, Lucy E. Wilson, Marco Garcia, Jennifer Zipprich, Marisa R. Hoffman, Kristina G. Flores, Julia Tellerman, Ghinwa Dumyati, Shannon O’Brien, Daniel B. Muleta, Olivia Denzie, Alice Y. Guh

**Affiliations:** 1 Division of Healthcare Quality Promotion, Centers for Disease Control and Prevention, Atlanta, Georgia; 2 California Emerging Infections Program, Oakland; 3 Colorado Department of Public Health and Environment, Denver; 4 Georgia Emerging Infections Program, Atlanta; 5 Emory University School of Medicine, Atlanta, Georgia; 6 Maryland Department of Health, Baltimore; 7 Baltimore County Department of Health and Human Services, Baltimore, Maryland; 8 Minnesota Department of Health, St Paul; 9 University of New Mexico, Albuquerque; 10 New York Rochester Emerging Infections Program at the University of Rochester Medical Center, Rochester; 11 Oregon Health Authority, Acute and Communicable Disease Prevention, Salem; 12 Tennessee Department of Health, Nashville

## Abstract

**Importance:**

Extraintestinal invasive *Escherichia coli* infections are a leading cause of sepsis and hospitalization, further complicated by increasing rates of antimicrobial resistance.

**Objective:**

To describe the US epidemiology of invasive *E coli* infections and their clinical and molecular features.

**Design, Setting, and Participants:**

This cohort study used active laboratory- and population-based surveillance data from 9 US sites with a total population of more than 7.2 million people for invasive *E coli* in normally sterile body sites, collected from June to August 2023 from medical records and isolate characterization. Data were analyzed from November 2023 to February 2024.

**Exposures:**

Laboratory-confirmed identification of any *E coli* organism isolated from a normally sterile body site obtained from a resident of the surveillance area.

**Main Outcomes and Measures:**

Outcomes of interest were population-based and site-specific incidence rate estimates of *E coli* infections, demographic and clinical characteristics, antimicrobial susceptibility profiles, and predicted O serotypes by in silico serotyping.

**Results:**

Among 1345 cases of *E coli* infection in 1334 unique case-patients, the median (IQR) age was 68 (55-79) years, and 762 case-patients (57.1%) were female; 1223 infections (90.9%) were from blood and 122 infections (9.1%) were from other sterile sites. The overall estimated annual incidence rate was 74.7 per 100 000 population (surveillance site range, 51.4-96.0 per 100 000 population). Estimated incidence rates were higher among cases in patients aged 60 years or older compared with younger patients (225.0 vs 30.6 per 100 000 population), although rates were similar for females and males aged 60 years or older (224.5 vs 224.0 per 100 000 population). The most common underlying medical condition reported was diabetes (457 patients [34.0%]). Pyelonephritis (267 infections [19.9%]) and lower urinary tract infections (495 infections [36.8%]) were the most frequently associated infection types. In total, 1279 cases (95.1%) were hospitalized for less than 30 days after isolate collection; 106 case-patients (7.9%) died. Overall, 185 infections (13.8%) were due to extended-spectrum β-lactamase-producing *E coli*; 275 of 1061 isolates (25.9%) were resistant to ciprofloxacin and 370 of 1286 (28.8%) were resistant to trimethoprim-sulfamethoxazole. Of 846 sequenced isolates, the most prevalent O serotypes were O25B (137 isolates [16.2%]), O2 (93 isolates [11.0%]), and O6 (84 isolates [9.9%]).

**Conclusions and Relevance:**

This cohort study using population-based public health surveillance data identified a substantial burden of invasive *E coli* disease, especially in older people, with high rates of antimicrobial resistance. These results can help inform national public health prevention efforts.

## Key Points

**Question:**

What are the incidences, antibiotic susceptibilities, and O serotypes of extraintestinal invasive *Escherichia coli* infections in US communities?

**Findings:**

In this cohort study using population-based data from more than 7.2 million people, there were 1345 invasive *E coli* infections; incidence rates were higher among older compared with younger populations and most frequently associated with infections of the urinary tract. Nearly 14% of infections were caused by extended-spectrum β-lactamase–producing *E coli*, and O25B was the most prevalent O serotype.

**Meaning:**

These findings suggest there is a significant burden of invasive *E coli* infections in US communities, and high rates of antimicrobial resistance in this pathogen warrant continued prevention efforts.

## Teaser

This cohort study uses population-based data to describe the incidence and features of extraintestinal invasive *Escherichia coli* infections in the US.

## Introduction

Extraintestinal invasive *Escherichia coli* is the most common gram-negative bacterial pathogen in humans, causing a diverse range of clinical disease outside of the gastrointestinal tract.^1,2,3^ It is a frequent cause of community-onset urinary tract infections (UTIs),^4,5^ bloodstream infections,^6^ neonatal meningitis,^7,8^ and hospital-acquired infections.^9,10^ In addition, invasive *E coli* is the most common pathogen responsible for community-onset sepsis in the US,^3,11^ a leading cause of death in hospitalized patients.^3^ Further characterizing the burden of invasive *E coli* infections in US populations has important implications for informing prevention strategies to improve sepsis detection and management and better protect the health of patients.

Not only have rates of invasive *E coli* infections increased globally,^10,12^ but treatment is increasingly complicated by the rise in antimicrobial resistance. This also creates major challenges for infection prevention and control.^13,14,15^ The US Centers for Disease Control and Prevention (CDC) categorizes third-generation cephalosporin-resistant and carbapenem-resistant Enterobacterales, including invasive *E coli*, as serious or urgent public health threats, respectively.^16^ Recent data from hospitalized patients in the US identified more than 34% of invasive *E coli* infections with resistance to 3 or more antibiotic classes,^17^ and multicountry studies have estimated more than 10% of *E coli* blood stream isolates were multidrug resistant.^18^ In addition, mobile genetic elements conferring antimicrobial resistance can be transferred from *E coli* to other pathogenic or opportunistic bacteria and further the spread of resistance.^19,20^

Previously completed large-scale epidemiologic studies of invasive *E coli* in the US are limited to older adults or hospitalized cohorts^14,17,21^ or lack corresponding serotype data.^13^ Despite its clinical importance, no routine public health surveillance has previously existed in the US for invasive *E coli* of any antimicrobial susceptibility phenotype. The CDC’s Emerging Infections Program (EIP) conducts active laboratory- and population-based surveillance for selected gram-negative organisms through the Multi-site Gram-negative Surveillance Initiative (MuGSI).^22^ Our objective was to describe the epidemiology of invasive *E coli* infections in the US, their antibiotic susceptibility profiles, and estimated O serotype distribution using data from a 3-month pilot of surveillance for invasive *E coli* infections conducted through EIP’s MuGSI network.

## Methods

This cohort study was reviewed by the CDC, deemed not to be human participant research, and was conducted consistent with applicable federal law and CDC policy (eg, 45 CFR. part 46.102[l][2]; 21 CFR part 56; 42 USC §241[d]; 5 USC §552a; 44 USC §3501). The protocol was reviewed by all participating EIP sites, and sites obtained ethics approval from their respective state health department and academic partner institutional review boards when applicable. The study was deemed nonresearch or received institutional review board approval with a waiver of informed consent at all sites. This study is reported following the Strengthening the Reporting of Observational Studies in Epidemiology (STROBE) reporting guideline.

### Surveillance Population

Population-based invasive *E coli* surveillance was conducted in selected metropolitan counties at 9 EIP sites (California, Colorado, Georgia, Maryland, Minnesota, New Mexico, New York, Oregon, and Tennessee) during June, July, and August 2023. The total population of the participating areas under surveillance was more than 7.2 million people (eMethods in Supplement 1).

### Case Definition and Data Collection

We defined an incident case of invasive *E coli* as the first isolation of *E coli* of any antimicrobial susceptibility profile from a normally sterile body site over a 30-day period from a resident of the surveillance area. Normally sterile sites included blood, cerebrospinal fluid, pleural fluid, pericardial fluid, peritoneal fluid, synovial fluid, bone, internal body tissue (lymph node, brain, heart, liver spleen, vitreous fluid, kidney, pancreas, or ovary), muscle, deep tissue, and other normally sterile sites. Cases were identified through a query of clinical laboratory automated testing instruments based on laboratory protocols. Incident invasive *E coli* cases underwent medical record review using a standardized case report form^22^ to collect patient demographics, underlying conditions, health care exposures and outcomes, location of specimen collection, associated infection types, and antimicrobial susceptibility testing results (eMethods in Supplement 1). Because risk of invasive *E coli* infection may vary by race and ethnicity, race and ethnicity were obtained from medical records as part of the demographic information. Race and ethnicity data collected directly from patient medical records were categorized by EIP staff using predefined categories in accordance with Office of Management and Budget standards (Hispanic or Latino, any race; not known to be Hispanic, American Indian; not known to be Hispanic, Asian; not known to be Hispanic, Black or African American; not known to be Hispanic, White; and not known to be Hispanic, another race [hereafter, Hispanic, American Indian, Asian, Black, White, and another race, respectively). Due to small case numbers or population denominators, data for Native Hawaiian patients, Pacific Islander patients, or patients who identified as multiple races were included in the another race category for privacy.

Underlying conditions were grouped such that cardiovascular disease includes any history of cerebrovascular accident, transient ischemic attack, congenital heart disease, congestive heart failure, myocardial infarction, or peripheral vascular disease. Chronic kidney disease includes any history of end-stage kidney disease with or without dialysis. Chronic lung disease includes any history of chronic pulmonary diseases with symptomatic dyspnea due to a chronic respiratory condition, such as chronic obstructive pulmonary disease, emphysema, interstitial lung disease, asthma, cystic fibrosis, and other conditions causing respiratory failure. Dementia includes chronic loss of intellectual abilities, such as memory capacity severe enough to interfere with social or occupational functioning, such as Alzheimer disease. Gastrointestinal disease includes any history of diverticular disease, inflammatory bowel disease, peptic ulcer disease, or short gut syndrome. Liver disease includes any history of chronic liver disease, chronic liver failure, or chronic hepatitis from any cause. Malignant neoplasm includes any history of hematologic or solid organ cancers. Neurologic condition includes any history of cerebral palsy, chronic cognitive deficit, dementia, epilepsy, multiple sclerosis, neuropathy, Parkinson disease, or others. Transplant includes both hematopoietic stem cell and solid organ. Urinary tract problems or abnormalities includes any history of a structural abnormality leading to obstruction or retention of urine, such as bladder prolapse, vesicoureteral reflux, recurrent kidney stones, or others.

### Isolate Characterization

Laboratories serving surveillance area residents were requested to submit as many incident case *E coli* isolates as possible to CDC for confirmatory testing and molecular characterization. Antimicrobial resistance profiles reported from local clinical labs were included on the case report form. We defined incident carbapenem-resistant (CR) *E coli* cases as those resistant to at least 1 carbapenem and extended-spectrum β-lactamase-producing (ESBL) *E coli* cases as those resistant to at least 1 extended-spectrum cephalosporin and nonresistant (ie, susceptible or intermediate) to all tested carbapenems. At CDC, isolates underwent whole-genome sequencing and in silico serotyping (eMethods in Supplement 1).

### Statistical Analysis

Three-month incidence rates were calculated by dividing the total invasive *E coli* cases by 2023 postcensal county population estimates. Annual incidence rates were estimated using incident case counts multiplied by 4 to approximate a year of surveillance, divided by 2023 postcensal county population estimates as the denominator.^23^ Incidence rates within selected population subgroups (such as age groups) were calculated using the numbers of cases in the subgroup divided by the total surveillance area population for the subgroup. Data were additionally stratified by 60 years or older and younger than 60 years to match age groups targeted by recent vaccine clinical trials^24^ and with higher frequency of invasive *E coli* sepsis.^11^ Outcomes were reported based on the status of the case-patient at discharge for all hospitalized case-patients. Mortality rates were calculated using mortality at hospital discharge as the numerator and case-patient counts as the denominator. Data analyses were conducted using SAS software version 9.4 (SAS Institute). Data were analyzed from November 2023 to February 2024.

## Results

During the pilot period, 1345 incident cases of *E coli* infection were identified from 1334 unique case-patients (median [IQR] age, 68 [55-79] years; 762 [57.1%] female), with most infections occurring among cases aged 60 years or older (919 infections [68.3%]) (Table 1). There were 22 cases (1.6%) among children, including 10 cases (0.7%) in children younger than 1 year. Most cases (1194 [88.8%]) were among patients with underlying medical conditions reported, including 457 infections (34.0%) among patients with diabetes, 405 infections (30.1%) among patients with neurologic conditions, and 403 infections (30.0%) among patients with cardiovascular disease (Table 1). Median (range) Charlson Comorbidity Index^25^ across all case-patients was 2 (0-16).

**Table 1.** Demographic and Clinical Characteristics of Patients With Incident Invasive *Escherichia coli* Infection by Age Group

Characteristic	Infections, No. (%)		
	Total (N = 1345)	Cases in patients aged <60 y (n = 426)	Cases in patients aged ≥60 y (n = 919)
Sex^a^			
Female	766 (57.0)	265 (62.2)	501 (54.5)
Male	570 (42.4)	155 (36.4)	415 (45.2)
Age group, y			
0-1	10 (0.7)	10 (2.3)	NA
1-17	12 (0.9)	12 (2.8)	NA
18-39	136 (10.1)	136 (31.9)	NA
40-59	268 (19.9)	268 (62.9)	NA
60-79	606 (45.1)	NA	606 (65.9)
≥80	313 (23.3)	NA	313 (34.1)
Race and ethnicity^b^			
Hispanic or Latino, any race	218 (16.2)	115 (27.0)	103 (11.2)
Not known to be Hispanic, American Indian	17 (1.3)	9 (2.1)	8 (0.9)
Not known to be Hispanic, Asian	125 (9.3)	37 (8.7)	88 (9.6)
Not known to be Hispanic, Black or African American	282 (21.0)	114 (26.8)	168 (18.3)
Not known to be Hispanic, White	641 (47.7)	126 (29.6)	515 (56.0)
Not known to be Hispanic, another race^c^	41 (3.0)	18 (4.2)	23 (2.5)
Underlying conditions^d^			
None	148 (11.0)	89 (20.9)	59 (6.4)
Cardiovascular disease	403 (30.0)	46 (10.8)	357 (38.8)
Chronic kidney disease	326 (24.3)	41 (9.6)	285 (31.0)
Chronic lung disease	275 (20.5)	63 (14.8)	212 (23.1)
Dementia	137 (10.2)	3 (0.7)	134 (14.6)
Diabetes	457 (34.0)	113 (26.5)	334 (36.3)
Gastrointestinal disease	143 (10.6)	27 (6.3)	116 (12.6)
HIV	26 (1.9)	13 (3.1)	13 (1.4)
Liver disease	92 (6.8)	40 (9.4)	52 (5.7)
Malignant neoplasm	230 (17.1)	43 (10.1)	187 (20.3)
Neurologic condition	405 (30.1)	77 (18.1)	328 (35.7)
Obesity	302 (22.5)	95 (22.3)	207 (22.5)
Pregnancy	10 (0.7)	10 (2.3)	0
Recurrent urinary tract infections	192 (14.3)	63 (14.8)	129 (14.0)
Transplant	41 (3.1)	17 (4.0)	24 (2.6)
Urinary tract problems or abnormalities	340 (25.3)	86 (20.2)	254 (27.6)
Any underlying condition	1194 (88.8)	335 (78.6)	859 (93.5)
Smoking (tobacco)	188 (14.0)	79 (18.5)	109 (11.9)
Alcohol abuse	87 (6.5)	48 (11.3)	39 (4.2)

Abbreviation: NA, not applicable.

^a^ Sex was not documented in the medical record for 9 cases (0.7%); 6 were <60 years (1.4%); 3 were ≥60 years (0.3%).

^b^ Race and ethnicity were not documented in the medical record for 21 cases (1.6%), including 7 (1.6%) younger than 60 years and 14 (1.5%) 60 years or older.

^c^ Due to small numbers or population denominators, data for Native Hawaiian patients, Pacific Islander patients, or patients who identified as multiple races were included in the another race category for privacy.

^d^ Underlying illnesses or prior medical history as documented in the medical record existing prior to hospitalization. Case-patients may have more than 1 underlying condition reported. Definitions of specific conditions appear in the Methods section.

### Incidence Rates

The overall 3-month crude incidence rate was 18.7 infections per 100 000 population (range, 12.0-24.0 infections per 100 000 population per EIP site), with an overall estimated crude annual incidence rate of 74.7 infections per 100 000 population (range, 51.4-96.0 infections per 100 000 population per EIP site) (Table 2). Estimated crude annual incidence rates were highest for females (83.4 infections per 100 000 population), Black individuals (79.5 infections per 100 000 population), and individuals aged 60 years or older (225.0 infections per 100 000 population). When stratified by age and sex, females had higher estimated crude annual incidence rates than males in younger age groups, especially in the age 18 to 39 years group (36.2 vs 10.8 infections per 100 000 population). This difference diminished for females and males age 60 years or older (224.5 vs 224.0 infections per 100 000 population).

**Table 2.** Number of Case-Patients, Incident Cases, and Estimated Crude Annual Incident Rates of Incident Invasive *Escherichia coli* Infection by Emerging Infections Program Site and Demographic Characteristics

Characteristic	No. (%)		Estimated crude annual incidence rate per 100 000 population^b^
	Case-patients^a^ (n = 1334)	Incident cases (n = 1345)	
Emerging infections program site			
California	345 (25.9)	349 (25.9)	86.1
Colorado	42 (3.1)	42 (3.1)	51.4
Georgia	155 (11.6)	157 (11.7)	58.2
Maryland	101 (7.6)	101 (7.5)	71.5
Minnesota	301 (22.6)	303 (22.5)	67.5
New Mexico	152 (11.4)	152 (11.3)	90.5
New York	156 (11.7)	158 (11.7)	84.4
Oregon	52 (3.9)	53 (3.9)	96.0
Tennessee	30 (2.2)	30 (2.2)	67.9
Total	1334 (100)	1345 (100)	74.7
Demographic characteristics			
Sex^c^			
Female	762 (57.1)	766 (57.0)	83.4
Male	563 (42.2)	570 (42.4)	64.5
Race^d^			
American Indian or Alaskan Native	19 (1.4)	20 (1.5)	76.1
Asian	127 (9.5)	128 (9.5)	55.7
Black or African American	282 (21.1)	285 (21.2)	79.5
White	720 (54.0)	724 (53.8)	64.9
Another race^e^	18 (1.3)	18 (1.3)	25.0
Age, y			
0-17	22 (1.6)	22 (1.6)	6.0
18-39	136 (10.2)	136 (10.1)	23.7
40-59	265 (19.9)	268 (19.9)	59.4
60-79	599 (44.9)	606 (45.1)	177.1
≥80	312 (23.4)	313 (23.3)	472.3
Subgroup by sex and age, y			
Female			
0-17	13 (1.0)	13 (1.0)	7.2
18-39	104 (7.8)	104 (7.7)	36.2
40-59	146 (10.9)	148 (11.0)	65.0
60-79	307 (23.0)	308 (22.9)	168.6
≥80	192 (14.4)	193 (14.3)	476.9
Male			
0-17	9 (0.7)	9 (0.7)	4.8
18-39	31 (2.3)	31 (2.3)	10.8
40-59	114 (8.5)	115 (8.6)	51.5
60-79	290 (21.7)	296 (22.0)	185.6
≥80	119 (8.9)	119 (8.8)	461.3

^a^ Unique patients with infections meeting incident case definition.

^b^ Estimated by multiplying pilot incident case counts by 4 to approximate 1 year total, divided by total population of site catchment by year and shown per 100 000 population.

^c^ Sex was not documented in the medical record for 9 cases (0.7%).

^d^ Race was not documented in the medical record for 170 cases (12.6%).

^e^ Due to small case numbers and/or population denominators, data for Native Hawaiian, Pacific Islander, or multiple race case-patients were included in the “another race” category for privacy.

Blood was the most common normally sterile specimen from which *E coli* was cultured; 1223 incident cases (90.9%) were identified from blood (estimated crude annual incidence rate, 67.9 infections per 100 000 population) and 122 incident cases (9.1%) were from other sterile sites (estimated crude annual incidence rate, 6.8 infections per 100 000 population). Of case-defining incident cultures, 200 infections (14.9%) were positive for at least 1 additional organism other than invasive *E coli*; of these 200 cultures, 148 (74.0%) were polymicrobial blood cultures.

### Associated Infection Types and Previous Health Care Exposures

Most cases had their incident cultures collected in an emergency department (1057 cultures [78.6%]) or inpatient setting (252 cultures [18.7%]) (Table 3). Culture-confirmed or clinically documented types of infection associated with the invasive *E coli* culture are also shown in Table 3. Following, and nonmutually exclusive of, bacteremia (1238 infections [92.0%]), pyelonephritis (267 infections [19.9%]) or lower UTIs (495 infections [36.8%]) were the most frequently associated infection types. More than 16% of cases were associated with septic shock (221 infections [16.4%]). When limiting to 1223 cases identified from a blood culture, 746 (61.0%) were associated with any UTI, 11 (0.9%) with peritonitis, and 8 (0.7%) with acute cholecystitis or cholangitis. Overall, review of health care exposures identified 546 invasive *E coli* cases (40.6%) were in patients with a history of acute care hospitalization in the year prior, and 154 cases (11.4%) occurred in residents of a long-term care facility.

**Table 3.** Specimen Collection Location, Associated Infection Types, Prior Health Care Exposures, Epidemiologic Classification, and Outcomes of Incident Invasive *Escherichia coli* Infections by Age Group

Characteristic	Infections, No. (%)		
	Total (N = 1345)	Cases in patients aged <60 y (n = 426)	Cases in patients aged ≥60 y (n = 919)
Isolate collection location			
Emergency department	1057 (78.6)	320 (75.1)	737 (80.2)
Inpatient	252 (18.7)	95 (22.3)	157 (17.1)
Outpatient	31 (2.3)	10 (2.3)	21 (2.3)
Long-term care facility	3 (0.2)	1 (0.2)	2 (0.2)
Long-term acute care facility	1 (0.1)	0	1 (0.1)
Unknown	1 (0.1)	0	1 (0.1)
Associated infection type^a^			
Bacteremia	1238 (92.0)	380 (89.2)	858 (93.4)
Catheter site infection	1 (0.1)	1 (0.2)	0
Cholecystitis or cholangitis	8 (0.6)	0	8 (0.9)
Peritonitis	36 (2.7)	18 (4.2)	18 (2.0)
Pneumonia	45 (3.3)	9 (2.1)	36 (3.9)
Pyelonephritis or lower UTI			
Overall	762 (56.7)	234 (54.9)	528 (57.5)
Pyelonephritis	267 (19.9)	133 (31.2)	134 (14.6)
Lower UTI	495 (36.8)	101 (23.7)	394 (42.9)
Surgical incision or site infection	16 (1.2)	6 (1.4)	10 (1.1)
Septic shock^b^	221 (16.4)	63 (14.8)	158 (17.2)
Health care exposures during prior year^c^			
Acute care hospitalization	546 (40.6)	154 (36.2)	392 (42.7)
Resident of long-term care facility	154 (11.4)	22 (5.2)	132 (14.4)
Admission to long-term acute care hospital	6 (0.4)	1 (0.2)	5 (0.5)
Inpatient or outpatient surgery	249 (18.5)	78 (18.3)	171 (18.6)
Chronic dialysis	46 (3.4)	14 (3.3)	32 (3.5)
Urinary catheter on day of collection or within 2 d prior	213 (15.8)	55 (12.9)	158 (17.2)
Indwelling urethral catheter	149 (11.1)	39 (9.2)	110 (12.0)
Suprapubic catheter	20 (1.5)	9 (2.1)	11 (1.2)
Central venous catheter on day of collection or within 2 d prior	102 (7.6)	45 (10.6)	57 (6.2)
Epidemiologic classification			
Hospital onset^d^	119 (8.8)	44 (10.3)	75 (8.2)
Health care–associated community onset^e^	633 (47.1)	172 (40.4)	461 (50.2)
Community associated^f^	554 (41.2)	200 (46.9)	354 (38.5)
Unknown	39 (2.9)	10 (2.3)	29 (3.2)
Hospitalization on DISC or in the 29 d after DISC	1279 (95.1)	387 (90.8)	892 (97.1)
ICU admission ≤6 d after DISC	296 (22.0)	95 (22.3)	201 (21.9)
Disposition at hospital discharge^g^			
Long-term acute care facility	10 (0.7)	4 (0.9)	6 (0.7)
Long-term care facility	245 (18.2)	29 (6.8)	216 (23.5)
Home (private residence)	911 (67.7)	321 (75.4)	590 (64.2)
Hospitalized and died	103 (7.7)	28 (6.6)	75 (8.2)
Other or unknown	11 (0.8)	5 (1.2)	6 (0.7)
Not hospitalized	65 (4.8)	39 (9.2)	26 (2.8)
Outcome^h^			
Survived	1235 (91.8)	396 (93.0)	839 (91.3)
Died	106 (7.9)	28 (6.6)	78 (8.5)
Unknown	4 (0.3)	2 (0.5)	2 (0.2)

Abbreviations: DISC, date of incident specimen collection; ICU, intensive care unit; UTI, urinary tract infection.

^a^ Culture-proven or clinically documented infection types associated with the incident *E coli* culture. Cases may have more than 1 associated infection type reported (eg, bacteremia and acute cholecystitis), shown independently. Cases with bacteremia indicated alone (332 cases [27.1%]) occurred when public health surveillance officers were unable to determine with certainty from the medical record what the treating medical team defined as the original source of bacteremia.

^b^ Identified based on medical record documentation only (eg, the phrase *septic shock* is included in a hospitalization history and physical or discharge summary).

^c^ Case-patients may have had more than 1 health care exposure during the prior year.

^d^ Defined as incident culture collected 3 or more days after hospital admission.

^e^ Defined as hospitalization, surgery, residence in a long-term care facility or long-term acute care hospital, or chronic dialysis in the year prior to culture, or indwelling device on day of culture or 2 days prior.

^f^ Defined as none of the health care risk factors previously described were identified.

^g^ Discharge disposition missing from 63 cases (5.1%) in completed case report forms (53 specimens from blood, 10 specimens from other sterile sites).

^h^ Outcome reported following first hospitalization for all hospitalized cases. For nonhospitalized case-patients, outcome reported when leaving care (eg, emergency department, clinical decision unit, observation unit).

### Hospitalizations and Outcomes

Among all cases, 1279 (95.1%) were hospitalized within 30 days after incident culture; 296 cases (22.0%) required intensive care unit admission (Table 3). Median (IQR) length of hospitalization was 5 (3-9) days. Most cases were discharged to a private residence (911 cases [67.7%]) or long-term care facility (245 cases [18.2%]). The crude in-hospital mortality rate among all case-patients was 7.9% (106 of 1334 case-patients); 59 deaths (55.7%) occurred in females, and 52 deaths (49.1%) occurred in White patients, followed by 32 Black patients (30.2%). Mortality rate was greater for cases with positive blood cultures compared with infections with cultures of other sterile site specimens (102 of 1213 infections [8.4%] vs 4 of 122 infections [3.3%]), and from those in case-patients aged 60 years or older (78 of 911 infections [8.6%]).

### Antimicrobial Resistance

The antimicrobial resistance profiles of isolates from incident invasive *E coli* infections reported from local clinical laboratories are shown in Table 4. Substantial resistance was reported for trimethoprim-sulfamethoxazole (370 of 1286 isolates [28.8%]), levofloxacin (194 of 720 isolates [26.9%]), ciprofloxacin (275 of 1061 isolates [25.9%]), ceftazidime (87 of 930 isolates [9.4%]), cefepime (80 of 949 isolates [8.4%]), and ceftriaxone (181 of 1173 isolates [15.4%]). Of 1345 isolates, 3 (0.2%) were carbapenem-resistant *E coli* were identified, all from blood cultures. Separately, 185 of 1345 cases (13.8%) were due to ESBL *E coli*, including 164 of 1223 isolates (13.4%) from blood cultures and 21 of 122 (17.2%) from cultures of other sterile site specimens.

**Table 4.** Antimicrobial Resistance of Incident Invasive *Escherichia coli* Infections Based on Reported Testing Results at Local Clinical Laboratories by Age Group

Antimicrobial agent^b^	No. of resistant isolates/total No. isolates reported (%)^a^		
	Overall	Patients aged <60 y	Patients aged ≥60 y
Aminoglycosides			
Amikacin	2/612 (0.3)	0/208	2/404 (0.5)
Gentamicin	130/1300 (10.0)	47/418 (11.2)	83/882 (9.4)
Tobramycin	121/820 (14.8)	39/252 (15.5)	82/568 (14.4)
Carbapenems			
Doripenem	1/86 (1.2)	1/26 (3.8)	0/60
Ertapenem	0/1338	0/424 (0)	0/914
Imipenem	1/205 (0.5)	1/59 (1.7)	0/146
Meropenem	2/1208 (0.2)	1/380 (0.3)	1/828 (0.1)
Cephalosporin or cephamycins			
Cefotaxime	29/160 (18.1)	10/47 (21.3)	19/113 (16.8)
Ceftazidime	87/930 (9.4)	24/300 (8.0)	63/630 (10.0)
Ceftriaxone	181/1173 (15.4)	63/368 (17.1)	118/805 (14.7)
Cefepime	80/949 (8.4)	29/307 (9.4)	51/642 (7.9)
Cefoxitin	26/218 (11.9)	11/71 (15.5)	15/147 (10.2)
Fluoroquinolones			
Ciprofloxacin	275/1061 (25.9)	88/333 (26.4)	187/728 (25.7)
Levofloxacin	194/720 (26.9)	60/217 (27.6)	134/503 (26.6)
β-lactam and non–β-lactam combination agents			
Amoxicillin-clavulanate	8/339 (2.4)	1/108 (0.9)	7/231 (3.0)
Piperacillin- tazobactam	51/1175 (4.3)	18/385 (4.7)	33/790 (4.2)
Ceftazidime-avibactam	0/32	0/10	0/22
Meropenem-vaborbactam	0/29	0/9	0/20
Imipenem-cilastatin-relebactam	0/0	0/0	0/0
Folate pathway antagonists			
Trimethoprim-sulfamethoxazole	370/1286 (28.8)	128/416 (30.8)	242/870 (27.8)
Other antimicrobials			
Aztreonam	48/486 (9.9)	18/181 (9.9)	30/305 (9.8)
Colistin	0/1	0/0	0/1
Fosfomycin	0/1	0/0	0/1
Nitrofurantoin	3/181 (1.7)	2/73 (2.7)	1/108 (0.9)
Tigecycline	1/104 (1.0)	0/37	1/67 (1.5)

^a^ Percentage reported represents the percentage of total cases with antibiotic susceptibility data reported on the case report form.

^b^ Organisms may have been resistant to more than 1 antimicrobial agent.

### O Serotype Prevalence and Distribution

Whole genome sequencing and in silico serotyping were performed for isolates from 846 cases (62.9%). Of the total isolate collection sequenced, the most prevalent O serotypes were O25B (137 isolates [16.2%]), O2 (93 isolates [11.0%]), O6 (84 isolates [9.9%]), O1A (62 isolates [7.3%]), O16 (54 isolates [6.4%]), and O75 (53 isolates [6.3%]). These 6 O serotypes represent 57.1% of all sequenced cases (Figure). Serotype O25B was the most frequent O serotype observed among blood isolates (132 of 786 isolates [16.8%]), including 32 of 259 cases (12.4%) occurring in patients younger than 60 years and 105 of 587 cases (17.9%) in patients 60 years or older, 70 of 484 cases (14.5%) in female patients and 67 of 354 cases (18.9%) in male patients, 78 of 489 cases (16.0%) with an associated upper or lower UTI, and 15 of 69 cases (21.7%) in patients who died (eTable in Supplement 1). After O25B, the most abundant serotype was O2, observed more commonly in isolates from other sterile sites (6 of 60 isolates [10.0%]) compared with O25B (5 of 60 isolates [8.3%]).

**Figure.**
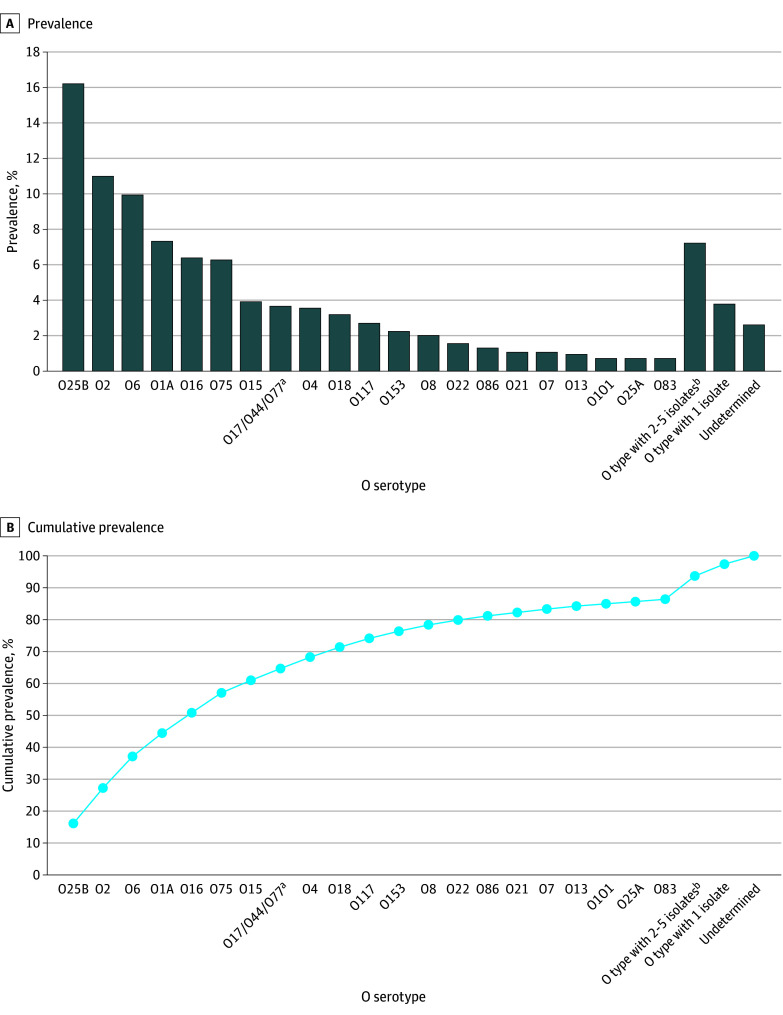
O Serotypes of Incident Invasive *Escherichia coli* Cases Based on Testing of Available Isolates Data from incident invasive *Escherichia coli* infections based on testing of 846 available isolates. From 9 Emerging Infections Program Sites, June-August 2023. ^a^Closely related O serotypes are reported as a group. ^b^O types not included in previously listed serotypes.

Of 185 ESBL-*E coli* isolates, sequencing and serotyping were performed for 88 isolates (47.6%); O25B was the most prevalent serotype (41 isolates [46.6%]), followed by O16 (8 isolates [9.1%]). Among isolates with antibiotic susceptibility data reported, O25B was the most frequent serotype among case isolates with resistance to ciprofloxacin (76 of 275 isolates [27.5%]), levofloxacin (67 of 194 isolates [34.5%]), and trimethoprim-sulfamethoxazole (36 of 370 isolates [9.7%]).

## Discussion

In this cohort study based on data collected from 9 diverse US geographic areas, we estimated an overall annual incidence rate for invasive *E coli* infections of 74.7 per 100 000 population (67.9 bloodstream infections per 100 000 population). The burden of these infections was higher in female case-patients and adults 60 years or older, and most infections were associated with an infection of the urinary tract. More than 95% of case-patients required hospitalization and nearly 8% died prior to hospital discharge.

These findings highlight the significant burden of invasive *E coli* infections and identify a higher estimated incidence rate in US communities than previously recognized. One multinational meta-analysis focused on *E coli* bacteremia previously estimated an incidence rate of 48 per 100 000 person-years in adults^6^; however, data on incidence rates from earlier publication years (2007–2018) were used in the review, and rates have increased over time.^26^ This increase is likely linked to several factors including an aging population, an age-associated increase in comorbidities and UTIs, and the emergence of multidrug-resistant strains (especially those that produce extended-spectrum β-lactamases).^27,28,29^ Our findings are similar to a 2021 multinational population-based study^30^ that identified an *E coli* bloodstream infection rate of 78.4 cases per 100 000 person-years in non-US populations. Our study used a culture-based case definition to identify invasive *E coli*. While this approach is specific, it likely underestimates incidence by missing cases of invasive *E coli* infections without positive bacterial cultures from sterile sites. Regardless, we found that the burden of invasive *E coli* infections and antimicrobial resistance was substantial, particularly when compared with rates of other invasive infections in the US, such as methicillin-resistant *Staphylococcus aureus* (19.0 infections per 100 000 person-years in 2021)^31^ and group A *Streptococcus* (8.2 infections per 100 000 person-years in 2022).^32^

Incidence of invasive *E coli* infection was different between sexes for younger age groups, especially among those aged 18 to 39 years age. UTIs from *E coli* result from bacteria ascending the urethra and are more common in females due to anatomical differences, especially at younger ages.^5,33^ Because urogenital infections accounted for more than half of associated infection types in our study, it was unsurprising to see a higher estimated incidence rate among younger females, reaffirming what has been previously observed.^6,34^

The population-based design of this surveillance data through EIP allowed us to capture both community-onset and hospital-onset invasive *E coli* cases. We observed that more than 88% of cases were community onset, consistent with previous data showing predominance of community-acquired *E coli* bloodstream infections.^13,30,35^ Despite most cases being community onset, nearly half had a health care exposure in the year prior to hospital admission. In addition, more than 40% of cases had acute care hospitalization reported in the year prior to invasive *E coli* infection. Initiatives aimed at improving sepsis outcomes, including sepsis caused by invasive *E coli* infections, may consider health care touchpoints as a potential opportunity for prevention interventions. This surveillance contributes to sepsis-related surveillance and prevention efforts led by the CDC, such as the Hospital Sepsis Program Core Elements^36^ and the National Healthcare Safety Network.^37^

Previous health care exposures may also contribute to the high rates of antimicrobial resistance observed. In our study, the highest prevalence of resistance was found for trimethoprim-sulfamethoxazole (28.8%), followed by levofloxacin (26.9%) and ciprofloxacin (25.9%). Trimethoprim-sulfamethoxazole is frequently used as an empiric treatment for uncomplicated UTIs, which are primarily caused by *E coli* in the outpatient setting. Additionally, fluoroquinolones remain a commonly used oral therapy for invasive *E coli* infections, and our findings further support the concerning increase in prevalence of fluoroquinolone resistance in *E coli*.^38,39^ These antimicrobial susceptibility percentages are comparable with those reported from other international and US studies,^13,14,35^ and they further emphasize the contribution of invasive *E coli* to the global antimicrobial resistance threat in both community and health care settings. Perhaps most concerning, nearly 14% of cases were associated with an ESBL *E coli* isolate. The high percentage of ESBL *E coli* identified from invasive infections is alarming and reflects recently published findings from the CDC EMERGEncy ID NET,^40^ which tracked patients hospitalized for urinary tract infections. The rise in ESBL *E coli* may give pause to clinicians making empiric treatment decisions for syndromes frequently caused by *E coli*, such as urosepsis.

These findings support evidence that O25B is the most prevalent O serotype in the US, which is consistent with previous studies.^18,41,42,43^ Based on in silico serotyping, we found that approximately 57% of invasive *E coli* cases were caused by 6 O serotypes, of which approximately 16% were O25B. Clinical trials targeting O antigens have been conducted for several different polysaccharide conjugate vaccine candidates against *E coli* infection, beginning as early as 1991.^44^ The most recent phase 3 clinical trial with a 9-valent conjugated polysaccharide vaccine carried out in adults 60 years and older could theoretically cover 67.5% of the total isolates sequenced from our collection and 77.3% of ESBL-*E coli* isolates sequenced.^24^ Vaccines targeting invasive *E coli* O serotypes could play an important role in a broader strategy to reduce antimicrobial resistance by preventing infections and reducing reliance on antibiotic treatment.

### Limitations

This study has several limitations inherent in the use of public health surveillance data. First, although the surveillance population was large, our findings may not be generalizable to the entire US population. Second, our case definition for invasive *E coli* infection relied on microbiologic identification for case ascertainment from sterile site cultures; because some infections may not have an associated culture due to factors such as timing of antibiotic administration prior to culture acquisition, it likely resulted in an underestimate of cases. In addition, sterile site cultures are discretionary and may not always be indicated at time of initial patient presentation. Third, data were retrospectively abstracted from medical records, and the quality of medical record documentation can vary between health care systems and facility types. Fourth, incidence rates for this 3-month pilot study were estimated by multiplying incident case counts by 4 to approximate 1 year. This method misses any seasonal variation occurring over time and is only an estimate of a full year of cases. Fifth, isolates were not sequenced for all infections and therefore may not be representative of all cases. Sixth, rather than agglutination O serotyping, isolates were sequenced and O serotypes were estimated based on in silico serotyping. For some isolates (2.6%), serotyping could not be estimated.

## Conclusions

In this cohort study using active population-based public health surveillance data of invasive *E coli* infection, we found significant burden of invasive *E coli* infections in US communities, occurring most frequently in people 60 years or older and most often associated with infections of the urinary tract. Notably, antimicrobial resistance identified in invasive *E coli* infections was substantial and remains an urgent public health concern, warranting continued attention and prevention resources. The surveillance network developed by the EIP for this pathogen contributes to an important gap in sepsis-related surveillance and prevention efforts. Our findings highlight the public health value of performing regular population-based surveillance and epidemiologic studies to inform prevention efforts, such as antimicrobial stewardship and vaccine development.

## Notes

Supplement 1.
eMethods.
**eTable. **Prevalence of O serotypes of incident invasive Escherichia coli cases based on testing of available isolates, 9 Emerging Infections Program Sites, June-August 2023
eReferences.

Supplement 2.
Data Sharing Statement
