# Error in Figure

**DOI:** 10.1001/jamanetworkopen.2026.4708

**Published:** 2026-03-06

In the Original Investigation titled “Extraintestinal invasive *Escherichia coli* infections in the US,”^1^ published February 2, 2026, the y-axis of Panel B in the Figure should have been labeled from 0 to 100 instead of 0 to 18. This article has been corrected.^1^
