# Supplementary material for: Extraintestinal Invasive Escherichia coli Infections in the US
Source: JAMA Netw Open. 2026 Feb 2;9(2):e2557201. doi: 10.1001/jamanetworkopen.2025.57201 (PMC12865657; doi:10.1001/jamanetworkopen.2025.57201)
Supplement: Supplement 1. — eMethods. eTable. Prevalence of O serotypes of incident invasive Escherichia coli cases based on testing of available isolates, 9 Emerging Infections Program Sites, June-August 2023 eReferences. [file jamanetwopen-e2557201-s001.pdf]

# Supplemental Online Content

Grome HN, Brandenburg JM, Kent AG, et al. Extraintestinal invasive *Escherichia coli* infections in the US. *JAMA Netw Open*. 2026;9(2):e2557201. doi:10.1001/jamanetworkopen.2025.57201

**eMethods.**

**eTable 1.** Prevalence of O serotypes of incident invasive Escherichia coli cases based on testing of available isolates, 9 Emerging Infections Program Sites, June-August 2023

**eReferences.**

This supplemental material has been provided by the authors to give readers additional information about their work.

© 2026 Grome HN et al. *JAMA Network Open*.

**eMethods**

*Surveillance Population*

The total population of the 9 participating areas for the invasive *E. coli* surveillance pilot activity in 2023 was an estimated 7.2 million people. Sections of metropolitan areas included as a part of the surveillance areas were Atlanta, GA (1 county, estimated population 1,079,105), Minneapolis/St. Paul, MN (2 counties, estimated population 1,794,788), Portland, OR (1 county, estimated population 220,768), Boulder, CO (1 county, estimated population 326,831), Baltimore City, MD (1 county, estimated population 565,239), Albuquerque, NM (1 county, estimated population 671,586), Rochester, NY (1 county, estimated population 748,482), south central TN (4 counties, estimated population 176,853), and San Francisco, CA (1 county, estimated population 1,622,188).

*Data Collection*

Invasive *E. coli* cases were identified through a query of clinical laboratory automated testing instruments based on the protocols of the laboratories. Antimicrobial susceptibility test methods varied among the clinical laboratories, although the majority reported the use of an automated test system (MicroScan™, Beckman Coulter, Inc.™ Diagnostics, Brea, CA; VITEK®, bioMérieux, Marcy-l'Étoile, FR, or BD Phoenix™, Becton, Dickinson and Company, Franklin Lakes, NJ). Kirby Bauer and E-tests were often used for confirmatory testing, if necessary, in antimicrobial resistant cases.

Incident invasive *E. coli* cases underwent medical record review using a standardized case report form, available online at the CDC Emerging Infections Program (EIP) Multisite Gram Negative Surveillance Initiative (MuGSI) website: https://www.cdc.gov/healthcare-associated-infections/php/haic-eip/mugsi.html. Medical record review is completed at each site by public health professionals trained specifically for clinical record abstraction. All abstractors undergo extensive EIP site-lead and CDC-lead training specific to the MuGSI case report form. In addition, the MuGSI case report form was not new to the invasive *E. coli* activity, and most staff have years of medical record review and data collection on this case report form as a part of the MuGSI EIP surveillance network. To ensure accuracy of reported data, data edits were completed by CDC on all submitted data to validate more complex variables (e.g., culture source, associated infection types). EIP site staff also completed chart reabstraction on some MuGSI cases to test for accuracy and agreement between staff.

*Whole Genome Sequencing*

Whole genome sequencing was conducted on isolates received at CDC. Sequencing was performed using an Illumina MiSeq or NovaSeq system (San Diego, CA). Genomic DNA was extracted using the Promega Maxwell 48 Low Elution Volume DNA Purification Kit and the Maxwell 48 MDx Instrument (Madison, WI, United States). Libraries were prepared from genomic DNA using Illumina DNA prep reagents (Illumina, San Diego, CA) and barcoding indices synthesized in the CDC Biotechnology Core Facility. The libraries underwent QC, normalization and pooling, and the final pool was sequenced using Illumina Novaseq 6000 SP Reagent kit v1.5 (500 cycles) (Illumina, San Diego, CA). Sequencing reads were filtered for read quality, basecalled and demultiplexed using bcl2fastq (v2.20).

All sequences were analyzed using the CDC laboratory's PHoeNIx pipeline (Portable Healthcare Nextgen Informatics; https://github.com/CDCgov/phoenix) to generate trimmed sequences and assemblies used for down-stream analyses.

*In silico Serotyping*

*In silico* O serotyping was conducted using two serotyping tools, ECTyper v1.0.0[1] and SerotypeFinder v2.0.2[2]. Each tool was run using both the assemblies and trimmed sequences for each isolate. The O-serotypes were identified as the highest scoring type from both tools. O25 and O1 subtypes were identified by comparing the assembly against an in-house constructed subtyping database using GAMMA v2.1[3].

© 2026 Grome HN et al. *JAMA Network Open*.

**eTable 1: Prevalence osf O serotypes of incident invasive Escherichia coli cases based on testing of available isolates, 9 Emerging Infections Program Sites, June-August 2023. (n = 846)**

| | Isolates, No. (% of sequenced) | | | | | | | | | |
|---|---|---|---|---|---|---|---|---|---|---|
| **Serotype** | **Total (%)** | **Blood (%)** | **Other sterile source (%)** | **Age <60 years (%)** | **Age ≥60 years (%)** | **Females (%)** | **Males (%)** | **Associated UTI (%)** | **ESBL-*E. coli* (%)** | **Died (%)** |
| **O25B** | 137 (16.2) | 132 (16.8) | 5 (8.3) | 32 (12.4) | 105 (17.9) | 70 (14.5) | 67 (18.9) | 78 (16.0) | 41 (46.6) | 15 (21.7) |
| **O2** | 93 (11.0) | 87 (11.1) | 6 (10.0) | 23 (8.9) | 70 (11.9) | 53 (11.0) | 38 (10.7) | 64 (13.1) | 3 (3.4) | 5 (7.2) |
| **O6** | 84 (9.9) | 81 (10.3) | 3 (5.0) | 27 (10.4) | 57 (9.7) | 46 (9.5) | 38 (10.7) | 53 (10.8) | 2 (2.3) | 9 (13.0) |
| **O1A** | 62 (7.3) | 60 (7.6) | 2 (3.3) | 0 (6.6) | 45 (7.7) | 40 (8.3) | 20 (5.6) | 44 (9.0) | 2 (2.3) | 7 (10.1) |
| **O16** | 54 (6.4) | 48 (6.1) | 6 (10.0) | 20 (7.7) | 34 (5.8) | 33 (6.8) | 21 (5.9) | 36 (7.4) | 8 (9.1) | 3 (4.3) |
| **O75** | 53 (6.3) | 49 (6.2) | 4 (6.7) | 19 (7.3) | 34 (5.8) | 36 (7.4) | 17 (4.8) | 29 (5.9) | 6 (6.8) | 5 (7.2) |
| **O15** | 33 (3.9) | 30 (3.8) | 3 (5.0) | 11 (4.2) | 22 (3.7) | 22 (4.5) | 10 (2.8) | 21 (4.3) | 4 (4.5) | 2 (2.9) |
| **O17/O44/O77**[a] | 31 (3.7) | 28 (3.6) | 3 (5.0) | 8 (3.1) | 23 (3.9) | 19 (3.9) | 1 (0.3) | 15 (3.1) | 1 (1.1) | 2 (2.9) |
| **O4** | 30 (3.5) | 26 (3.3) | 4 (6.7) | 12 (4.6) | 18 (3.1) | 14 (2.9) | 16 (4.5) | 20 (4.1) | 0 (0.0) | 2 (2.9) |
| **O18** | 27 (3.2) | 23 (2.9) | 4 (6.7) | 13 (5.0) | 14 (2.4) | 10 (2.1) | 10 (2.8) | 12 (2.5) | 2 (2.3) | 2 (2.9) |
| **O117** | 23 (2.7) | 23 (2.9) | 0 (0.0) | 13 (5.0) | 10 (1.7) | 17 (3.5) | 6 (1.7) | 14 (2.9) | 0 (0.0) | 0 (0.0) |
| **O153** | 19 (2.2) | 19 (2.4) | 0 (0.0) | 8 (3.1) | 11 (1.9) | 14 (2.9) | 5 (1.4) | 14 (2.9) | 2 (2.3) | 1 (1.4) |
| **O8** | 17 (2.0) | 16 (2.0) | 1 (1.7) | 4 (1.5) | 13 (2.2) | 8 (1.7) | 9 (2.5) | 7 (1.4) | 1 (1.1) | 0 (0.0) |
| **O22** | 13 (1.5) | 11 (1.4) | 2 (3.3) | 2 (0.8) | 11 (1.9) | 6 (1.2) | 7 (2.0) | 6 (1.2) | 0 (0.0) | 2 (2.9) |
| **O86** | 11 (1.3) | 10 (1.3) | 1 (1.7) | 6 (2.3) | 5 (0.9) | 7 (1.4) | 4 (1.1) | 5 (1.0) | 2 (2.3) | 0 (0.0) |
| **O21** | 9 (1.1) | 9 (1.1) | 0 (0.0) | 2 (0.8) | 7 (1.2) | 7 (1.4) | 2 (0.6) | 3 (0.6) | 0 (0.0) | 2 (2.9) |
| **O7** | 9 (1.1) | 8 (1.0) | 1 (1.7) | 3 (1.2) | 6 (1.0) | 8 (1.7) | 1 (0.3) | 5 (1.0) | 0 (0.0) | 1 (1.4) |
| **O13** | 8 (0.9) | 6 (0.8) | 2 (3.3) | 3 (1.2) | 5 (0.9) | 5 (1.0) | 3 (0.8) | 3 (0.6) | 0 (0.0) | 1 (1.4) |
| **O101** | 6 (0.7) | 5 (0.6) | 1 (1.7) | 2 (0.8) | 4 (0.7) | 3 (0.6) | 3 (0.8) | 2 (0.4) | 0 (0.0) | 0 (0.0) |
| **O25A** | 6 (0.7) | 5 (0.6) | 1 (1.7) | 1 (0.4) | 5 (0.9) | 3 (0.6) | 2 (0.6) | 5 (1.0) | 0 (0.0) | 0 (0.0) |
| **O83** | 6 (0.7) | 5 (0.6) | 1 (1.7) | 2 (0.8) | 4 (0.7) | 3 (0.6) | 3 (0.8) | 4 (0.8) | 2 (2.3) | 0 (0.0) |
| **O-type with 2–5 isolates**[b] | 61 (7.2) | 56 (7.1) | 0 (0.0) | 6 (5.8) | 39 (6.6) | 22 (4.5) | 39 (11.0) | 13 (2.7) | 6 (6.8) | 0 (0.0) |
| **O-type with 1 isolate**[b] | 32 (3.8) | 29 (3.7) | 8 (13.3) | 9 (4.2) | 28 (4.8) | 24 (5.0) | 25 (7.1) | 26 (5.3) | 6 (6.8) | 7 (10.1) |
| **O serotype undetermined** | 22 (2.6) | 20 (2.5) | 2 (3.3) | 0 (1.9) | 17 (2.9) | 14 (2.9) | 7 (2.0) | 10 (2.0) | 0 (0.0) | 3 (4.3) |
| **Total Sequenced / Total Incident Cases (%)** | 846 / 1345 (62.9) | 786 / 1223 (64.3) | 60 / 122 (49.2) | 259 / 426 (60.8) | 587 / 919 (63.9) | 484/766 (63.2) | 354/570 (62.1) | 489/762 (64.2) | 88/185 (47.6) | 69/106 (65.1) |

a Closely related O serotypes are reported as a group
b Of O types not listed above

© 2026 Grome HN et al. *JAMA Network Open*.

**eReferences**


1. Bessonov K, Laing C, Robertson J, et al. ECTyper: in silico Escherichia coli serotype and species prediction from raw and assembled whole-genome sequence data. Microb Genom **2021**; 7(12).
2. Joensen KG, Tetzschner AM, Iguchi A, Aarestrup FM, Scheutz F. Rapid and Easy In Silico Serotyping of Escherichia coli Isolates by Use of Whole-Genome Sequencing Data. J Clin Microbiol **2015**; 53(8): 2410-26.
3. Stanton RA VN, de Man TJB, Lawsin A, Halpin AL. Development and application of QuAISAR-H: A bioinformatics pipeline for short read sequences of health care-associated pathogens. In: ASM Conference on Rapid Applied Microbial Next Generation Sequencing and Bioinformatic Pipelines. Tyson Falls, 2022.


© 2026 Grome HN et al. *JAMA Network Open*.
