# Supplementary material for: Extraintestinal Invasive Escherichia coli Infections in the US
Source: JAMA Netw Open. 2026 Feb 2;9(2):e2557201. doi: 10.1001/jamanetworkopen.2025.57201 (PMC12865657; doi:10.1001/jamanetworkopen.2025.57201)
Supplement: Supplement 2. — Data Sharing Statement [file jamanetwopen-e2557201-s002.pdf]

Data Sharing Statement

Grome. Extraintestinal Invasive Escherichia coli Infections in the US. *JAMA Netw Open*. Published February 02, 2026. doi:10.1001/jamanetworkopen.2025.57201

Data
Data available: No

Additional Information
Explanation for why data not available: Individual patient data which is potentially identifiable will not be made available due to U.S. government requirements to protect patient privacy.
